# Construct Validity and Reliability of the Adult Rejection Sensitivity Questionnaire: A Comparison of Three Factor Models

**DOI:** 10.1155/2014/972424

**Published:** 2014-07-06

**Authors:** Marco Innamorati, Michela Balsamo, Beth Fairfield, Mariantonietta Fabbricatore, Antonino Tamburello, Aristide Saggino

**Affiliations:** ^1^Department of Psychological, Humanistic and Territorial Sciences (DISPUTer), “G. d'Annunzio” University, 66013 Chieti, Italy; ^2^European University of Rome, 00163 Rome, Italy; ^3^Skinner Institute, 00184 Rome, Italy

## Abstract

*Objectives and Methods*. The aim of the study was to investigate the construct validity of the ARSQ. *Methods*. The ARSQ and self-report measures of depression, anxiety, and hopelessness were administered to 774 Italian adults, aged 18 to 64 years. *Results*. Structural equation modeling indicated that the factor structure of the ARSQ can be represented by a bifactor model: a general rejection sensitivity factor and two group factors, expectancy of rejection and rejection anxiety. Reliability of observed scores was not satisfactory: only 44% of variance in observed total scores was due to the common factors. The analyses also indicated different correlates for the general factor and the group factors. *Limitations*. We administered an Italian version of the ARSQ to a nonclinical sample of adults, so that studies which use clinical populations or the original version of the ARSQ could obtain different results from those presented here. *Conclusion*. Our results suggest that the construct validity of the ARSQ is disputable and that rejection anxiety and expectancy could bias individuals to readily perceive and strongly react to cues of rejection in different ways.

## 1. Introduction

Rejection sensitivity can be considered a defensive motivation system activated in interpersonal contexts, which biases individuals to readily perceive and strongly react to cues of rejection [1]. Individuals with higher rejection sensitivity typically feel insecure and unhappy about their relationships and tend to perceive ambiguous behaviors in significant others as intentional rejection. On the one hand, rejection sensitivity predisposes individuals to react with more hostility and aggressiveness [2] and, on the other hand, to be more submissive in order to be accepted by someone who is considered important [3]. When the individual with higher rejection sensitivity fails to prevent rejection, he tends to react with self-directed hostile cognitions [4] and the development of depressive disorders [5, 6].

In line with their expectancy-value model of anxious expectations of rejection, Downey and Feldman [1] considered rejection sensitivity as the result of the interaction between rejection anxiety and expectancy of rejection and developed the Rejection Sensitivity Questionnaire (RSQ). This measure asks respondents to indicate their degree of concern and anxiety about the outcomes of 18 situations (e.g., “*How concerned or anxious would you be over whether or not your friend would want to help you out?*”), and their expectations of acceptance/rejection in such situations (e.g., “*I would expect that he/she would want to help me out*”) [1]. Importantly, this measure has also been adapted and administered to specific populations, such as children and adolescents (Child Rejection Sensitivity Questionnaire) [7] and adults (Adult Rejection Sensitivity Questionnaire) [8], since some situations in the original RSQ were very specific for undergraduate students. In particular, the Adult Rejection Sensitivity Questionnaire (ARSQ) [8, 9] was developed by revising situations described in the original version of the RSQ in order to adopt a more generally applicable wording, removing those situations specific to college life, and generating additional items about potential rejection situations in typical adult lives.

To date, the ARSQ has been used only in few studies with US samples [10, 11], and little information is available on its psychometric properties. Downey et al. [9] reported that the ARSQ correlated strongly with the original RSQ (*r* = 0.87) and had sufficient reliability (Cronbach alpha = 0.74). Berenson et al. [8] administered the ARSQ to 685 adults in an Internet survey and reported that, controlling for education, the ARSQ showed moderate associations with measures of neuroticism (*r* = 0.32), social avoidance/distress (*r* = 0.34), self-esteem (*r* = 0.46), attachment anxiety (*r* = 0.48) and avoidance (*r* = 0.33), interpersonal sensitivity (*r* = 0.45), and depression (*r* = 0.37). The authors also reported that the validity of the ARSQ was supported by its ability to reflect the individual differences in rejection sensitivity associated with serious forms of psychopathology in which rejection concerns are prominent [11].

However, to date, the psychometric properties of the ARSQ have not been fully investigated; for example, no studies assessed its dimensionality. The authors referred to the original 18-situation RSQ for the procedure of scoring and implicitly accepted the results of studies investigating the dimensionality of the RSQ as valid. However, to the best of our knowledge, the dimensionality of the RSQ is essentially based on the original study by Downey and Feldman [1], who first calculated RSQ composite scores by weighting the expected likelihood of rejection and the degree of concern over its occurrence and then submitting these composite scores to a principal component analysis. The authors interpreted their results as support in favor of the unidimensionality of the questionnaire, although the analyses performed on the whole sample and separately for each sex resulted in some factor loadings in the range 0.30–0.40, suggesting a less than strong homogeneity of content [1].

**Figure 1:**
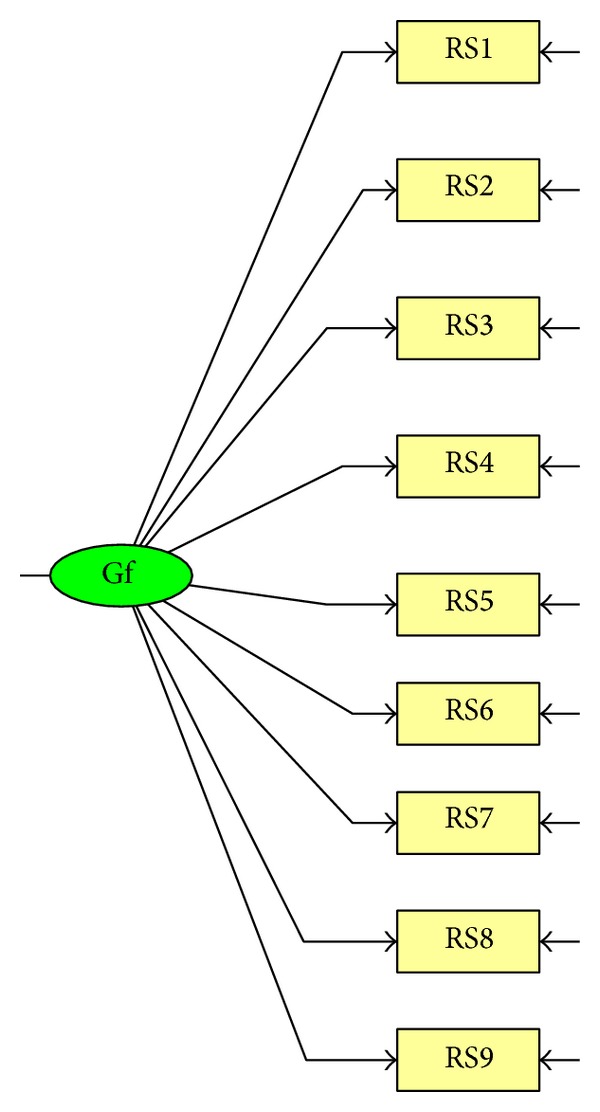
One-factor model.

**Figure 2:**
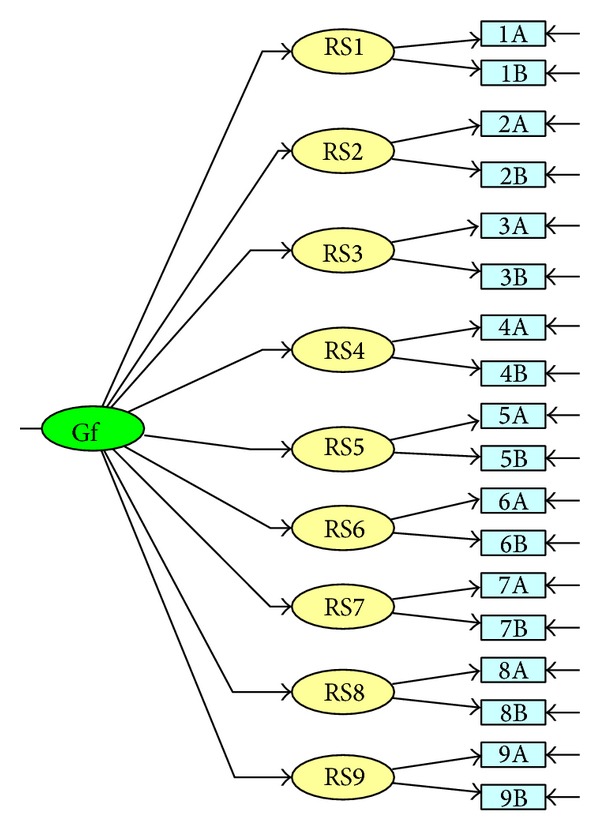
2nd-order factor model.

**Figure 3:**
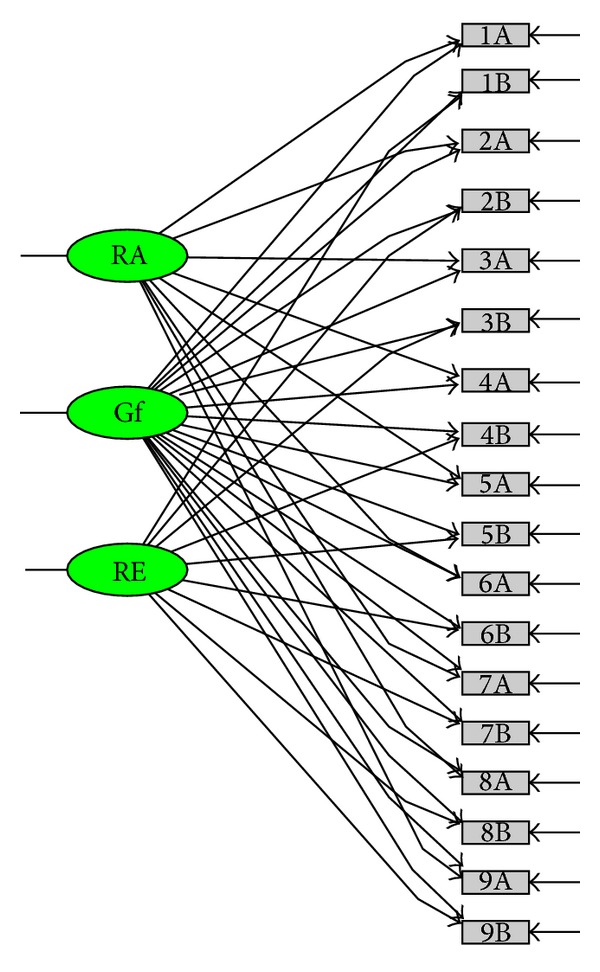
Bifactor model.

Thus, the evidence supporting the use of the ARSQ in the adult population is scarce, and information on the validity of this questionnaire is still limited. Thus, the major aim of the present research was to investigate construct validity of the ARSQ. First, we investigated the dimensionality of the ARSQ, comparing the original one-factor model (Figure 1) investigated by Downey and Feldman [1] in the original study on the RSQ with two alternative models (a 2nd-order factor model (Figure 2) and a bifactor model (Figure 3)). Furthermore, the one-factor model assumes that rejection expectancy and rejection concern contribute equally to the final rejection sensitivity score. The 2nd-order factor model (compared to the one-factor model) has the advantage that it tests the assumption that rejection sensitivity scores could be derived from weighting, for each situation, the expected likelihood of rejection and the degree of concern over its occurrence. Similarly, compared with the one-factor model, the bifactor model provides other advantages, permitting to address some questions still unexplored. (1) Is the ARSQ unidimensional or multidimensional? (2) To what degree does total scale score reflect reliable variation on a single construct? (3) Could rejection anxiety and expectancy be considered as two independent dimensions?

The bifactor model supposes the existence of a general factor explaining the covariance shared by all items and two specific factors explaining the covariance of single groups of items (groups of items assessing either anxiety for potential rejection or expectancy of rejection in each situation). Within the inspection of factor loadings of the bifactor model, if all items load strongly on the general factor and moderately on the group factors, evidence supporting a “quasi-unidimensionality” of the ARSQ is supported. On the contrary, if factor loadings for the group factors are strong, evidence for the multidimensionality of the ARSQ is supported.

Second, for the best fitting model we used McDonald's omega coefficients [12] to estimate reliability of the ARSQ and the proportion of the ARSQ score variance attributable to each common factor. Last, we investigated convergent validity with well-established measures of psychopathology (anxiety, depression, and hopelessness). Consistent with results reported by Berenson et al. [8], we expected the ARSQ to be positively and significantly associated with these convergent measures. Discriminant validity of the ARSQ and its subscales was examined comparing the pairs of correlation coefficients through the approach recommended by Meng et al. [13].

## 2. Materials and Methods

### 2.1. Participants and Procedure

We recruited 774 Italian adults (334 men and 430 women; mean age: 31.83 ± 14.45 years; age range: 18–64 years), between January 2011 and May 2011 in Central and Southern Italy. Sociodemographic characteristics of the sample are reported in Table 1. Inclusion criteria included age between 18 and 64 years and the ability to read and write in Italian. Exclusion criteria included the presence of any condition affecting the ability to complete the assessment, including illiteracy and denial of informed consent. The sample was recruited through attendants of adult education classes and an advertisement posted for established community groups. All participants voluntarily took part in the study, gave informed consent, and completed the assessment anonymously.

### 2.2. Measures

All participants were administered the ARSQ and validated Italian versions of the Beck Depression Inventory-II (BDI-II) [14], the Beck Hopelessness Scale (BHS) [15], and the Beck Anxiety Inventory (BAI) [16].

The ARSQ is an 18-item inventory specifically devised to measure rejection sensitivity in an adult population. The ARSQ consists of nine hypothetical situations involving interactions with partners, family, friends, and strangers, with potential rejection (e.g., “*You approach a close friend to talk after doing or saying something that seriously upset him/her,*” “*You ask your parents or another family member for a loan to help you through a difficult financial time,*” “*You bring up the issue of sexual protection with your significant other and tell him/her how important you think it is*”). Respondents are asked to rate the degree to which they are concerned or anxious over their reaction and the expectancy to be rejected on a 6-point Likert-type scale (ranging from 1, “*very unconcerned,*” to 6, “*very concerned,*” and from 1, “*very unlikely,*” to* 6, “very likely*”). The Italian version of the ARSQ was translated from the original English version by two authors of the present study (MI, MB); the resulting Italian version was then independently and blindly back translated by a native English speaking researcher.

The BDI-II is a well-known self-report inventory composed of 21 items designed to assess the presence and severity of depressive symptoms, according to DSM-IV [17] criteria. Respondents endorse specific statements reflecting their feelings over the last two weeks, including today. Each statement is rated on a 4-point Likert-type scale ranging from 0 to 3, based on the severity of depressive symptoms. Importantly, extensive literature supports the psychometric properties of the scale in clinical and nonclinical samples for the Italian version of the scale as well [18–20].

The BHS is a 20-item scale that measures negative attitudes about the future [15]. When responding to the 20 true-false items on the BHS, individuals either endorse a pessimistic statement or deny an optimistic statement. Research has consistently supported a positive significant relationship between BHS scores and measures of depression, suicidal intent, and suicidal ideation [21–25]. Studies on the Italian version of the BHS have been carried out successfully [26–28] and have led to validation of the scale [29].

The BAI is a 21-item self-report scale developed for measuring the severity of anxiety symptoms (e.g., “*wobbliness in legs*” and “*fear of losing control*”). Respondents have to rate how much they have been bothered by each symptom over the past week on a 4-point Likert-type scale, ranging from 0 (*not at all*) to 3 (*severely*). Past research has reported good psychometric properties for the original [30–35] and the Italian version of the BAI as well [36, 37].

### 2.3. Statistical Analysis

In line with our overall aim of determining the dimensionality of the ARSQ, we compared three different models.

1. A one-factor model (Figure 1) that is the model suggested from the study of Downey and Feldman [1] and supported by the authors of the ARSQ [8, 9]: in this model nine manifest variables, derived by weighing the response of the respondent on the expected likelihood of rejection for the response of the respondent on the degree of concern over its occurrence for each situation, load on a single latent factor.
2. A 2nd-order factor model (Figure 2): despite the fact that this model has never been investigated previously, it may fully represent the model of rejection sensitivity supported by Downey and Feldman [1]: in the left part of the diagram, manifest variables, obtained directly from the respondent's response to each item, load on 9 first-order rejection sensitivity factors, and in the right part of the diagram these factors load on a second-order latent common factor. We investigated this model, because the one-factor model and the other models included in our analyses have different manifest variables, and it is difficult to compare the fit of the competing models because of the different set of manifest variables used.
3. A bifactor model (Figure 3) [38]: in this model, each item loads on a general factor and a group factor [39]. The general factor explains the covariance shared by all items. The group factors account for the covariance independent of the general factor. The general factor and group factors are uncorrelated and account for the covariance simultaneously and independently for each item.

Due to the ordinal nature of the ARSQ items, we performed analyses with a Robust Diagonally Weighted Least Squares estimator (DWLSE) on a polychoric correlational matrix. All analyses were performed using the statistical package Lisrel 8.8 [40]. We evaluated the fit of the model by means of the following indeces.

- The Root Mean Square Error of Approximation (RMSEA): Browne and Cudeck [41] indicated values between 0.05 and 0.08 as indicators of acceptable fit, and values lower than 0.05 as indicators of good fit of the model tested. Hu and Bentler [42] recommended values lower than 0.06.
- The Incremental Comparative Fit Index (CFI): values higher than 0.95/0.96 indicate a good model fit [42].
- The Satorra-Bentler Scaled Chi-Square (*χ* ^2^): nonsignificant values indicate good model fit. Nevertheless, this index is dependent on sample size; thus, it always tends to be significant in large samples [43].
- The Standardized Root Mean Square Residual (SRMR): this is an index of absolute fit. It indicates a perfect fit of the model when values are close to zero. Hu and Bentler [42] suggested values lower than 0.08 as index of satisfactory model fit.

Cronbach alpha [44], mean interitems correlation, and corrected item-total correlations indices for the models that satisfactorily fit data were reported. Furthermore, we hand-calculated McDonald's omega, omega H, and omega S indices [45], according to the procedures suggested in Reise et al. [46]. We reported the omega index since the Cronbach alpha is considered a poor index of unidimensionality by some authors [47], when assumptions of the essentially tau-equivalent measurement model are not met (each item measures the same latent variable, on the same scale, with possibly different amounts of error and different degrees of precision) [48, 49]. On the contrary, McDonald's omega is based on the less restrictive congeneric model, which differs from other models because it assumes that each item measures the same latent variable with possibly different scales [48, 50]. Only when the assumptions of the essentially tau-equivalent measurement model are met, omega and alpha values are equals. Omega (the proportion of total score variance that can be attributed to all factors, group and general factors), omega H (the proportion of total score variance attributable to the general factor), and omega S (the proportion of total score variance that can be attributed to all group factors, after controlling for the general factor) were reported as estimates of the percentage of variance in observed scores due to variance on the single general factor (i.e., rejection sensitivity) and group factors (i.e., rejection anxiety and expectancy).

Finally, in order to explore the convergent validity of the ARSQ with measures of psychopathology, we measured the correlations of previously extracted factors with the BDI-II, the BHS, and the BAI total scores. We used the approach recommended by Meng et al. [13] to examine discriminant validity of the ARSQ and its subscales. This procedure involves performing a Fisher *Z* transformation on the correlation coefficients so that they can be compared via a *t*-test.

## 3. Results

### 3.1. Models Fit and Assessment

Fit statistics for the alternative SEM models are reported in Table 2. The analysis indicated that neither the original one-factor model nor the 2nd-order factor model fitted the data, while the bifactor model had adequate fit to the data (RMSEA = 0.077; 90% CI: 0.071/0.083; SRMSR = 0.066), supporting the presence of a general factor (Rejection Sensitivity—ARSQ-RS) and two group factors (Rejection Anxiety—RA, which includes the 9 items assessing anxiety for rejection, and Rejection expectancy—RE, which includes the 9 items assessing expectancy of rejection).

When comparing factor loadings of the group factors and those of the general factor, RE loadings were all greater than the corresponding loadings on the general factor, while only 4 of 9 RA loadings were greater than the corresponding loadings on the general factor (see Table 3). Furthermore, some residuals were large enough (>2.58) [51] to suppose that the bifactor model might not satisfactorily estimate the relationships between some pairs of variables.

### 3.2. Psychometric Properties of the Bifactor Model

Reliabilities and descriptive statistics for all measures administered are reported in Table 4. Although internal consistency for the general and the group factors was satisfactory, reliability as measured with McDonald's omega was not: only 44% of variance in observed total scores was due to the common factors (McDonald's omega = 0.44), and only 28% was due to the general factor (McDonald's omega H = 0.28). Sixty-five percent of the reliable variance in ARSQ scores was due to the general factor and 35% to RA and RE.

For part A (composed of the 9 items loading on RA) of the scale, 59% of variance in observed scores was due to the common factors (McDonald's omega = 0.59), and only 18% was due to RA (McDonald's omega S = 0.18). That means that only 30% of the reliable variance in observed scores was due to RA independently of the general factor. For part B (composed of the 9 items loading on RE) of the scale, 45% of variance in observed scores was due to the common factors (McDonald's omega = 0.45), and 34% was due to RE (McDonald's omega S = 0.34), so that 74% of the reliable variance in observed scores was due to RE independently of the general factor. The general factor and both the group factors correlated significantly and positively with convergent measures of depression, anxiety, and hopelessness, so that individuals with higher rejection sensitivity reported more depressive and anxiety symptoms and were more hopeless (Table 5). The correlations of the general factor with the BDI and the BAI were comparable to those of RA (*Z* = 0.54; *P* = 0.30 for the BDI; *Z* = 0.00; *P* = 0.50 for the BAI), except for the correlation with the BHS (*Z* = 2.62; *P* < 0.01), and were greater than those of RE (*Z* = 7.46; *P* < 0.001 for the BDI; *Z* = 5.05; *P* < 0.001 for the BAI; *Z* = 3.48; *P* < 0.001 for the BHS). The correlations of RA with measures of psychopathology were greater than those of RE (*Z* = 4.18; *P* < 0.001 for the BDI; *Z* = 3.00; *P* < 0.001 for the BAI) with the exclusion of the correlation with the BHS (*Z* = 0.82; *P* = 0.21).

## 4. Discussion

Our results suggest that the construct validity of the ARSQ is disputable. The unidimensional model proposed by Downey and Feldman [1] and supported by the authors of the ARSQ, as well as the alternative 2nd-order factor model, did not fit the data. Factor structure of the ARSQ may be represented by a bifactor model with a general rejection sensitivity factor and two group factors, rejection expectancy and rejection anxiety. Furthermore, the underlying hypothesis of the bifactor model according to which all the items will load strongly on the general factor and moderately on the group factors, so that the structure of the instrument could be considered mostly unidimensional, was not supported by these results. The pattern of factor loadings indicated that items, especially those assessing expectancy of rejection, loaded more strongly on the specific factors than on the general factor.

One other issue arising from the present study concerns the reliability of the ARSQ. Although internal consistency of group factors and the general factor was sufficient as indicated by alpha coefficients ranging between 0.78 and 0.82, omega coefficients showed low reliability of the scores of the ARSQ. This suggests that the assumptions of the essentially tau-equivalent measurement model were not met and indicates that the ARSQ total scores were not able to capture well the latent common variable they intend to measure. Furthermore, omega S coefficients seemed to suggest that rejection expectancy is a separate dimension of rejections sensitivity, and its specific roles in measuring rejection sensitivity should be more properly investigated.

Our pattern of correlations with convergent measures of psychopathology is consistent with the results reported by Berenson et al. [8], who indicated that ARSQ scores were moderately associated with neuroticism, social avoidance and distress, attachment anxiety and avoidance, interpersonal sensitivity, and depression, and is also consistent with the more vast literature on rejection sensitivity [6, 9, 11, 52–57]. However, these analyses also indicated different correlates of the general factor and the group factors, especially for rejection expectancy when compared with the general factor or rejection anxiety. On the contrary, rejection anxiety had different correlates than those of the general factor only for hopelessness. These results are not surprising if we consider that only 30% of the reliable variance in rejection anxiety observed scores was independent of the general factor.

Our study has limitations and strengths. Given that we used an Italian version of the ARSQ, results with the original version of the scale could be different. Furthermore, we only used self-reported measures which could be biased by social desirability [58, 59]. Nonetheless, an asset of the study is its breadth of sample. Additionally, as far as we know, this is the first study to investigate the dimensionality of the ARSQ. In line with the expectancy-value model of anxious expectations of rejection, Downey and Feldman [1] and the authors of the ARSQ [8, 9] always investigated the dimensionality of their measures by using “composite scores,” derived by weighting scores on items measuring the expected likelihood of rejection by scores on items measuring the degree of concern over its occurrence for each situation as measured variables. In our study, we compared the single-factor model with “composite scores” as measured variables with two other models that used items assessing the expected likelihood of rejection and items measuring the degree of concern over its occurrence as measured variables. Accordingly, we calculated rejections sensitivity scores by summing up all the items of the ARSQ, while in their research the authors of the ARSQ calculated rejections sensitivity scores summing up “composite scores.” A further limitation is that these differing procedures may produce scores with differing properties, and, therefore, future studies will have to compare the pros and cons of these two models of scoring. Last, our sample was extracted from a nonclinical population; thus, the assessment of the factor structure of the ARSQ may produce different results in clinical populations.

In conclusion, our findings raise questions about the construct validity of the ARSQ. In our nonclinical sample, the ARSQ appears not able to capture well the general dimension of rejection sensitivity. Furthermore, rejection anxiety and expectancy could bias individuals to readily perceive and strongly react to cues of rejection in different ways. Thus, caution should be used in interpreting the results from those studies which used the ARSQ, and in interpreting the expectancy-value model of anxious expectations of rejection. However, future research is needed and should focus on the study of the psychometric characteristics of the original version of the ARSQ in clinical and nonclinical populations. Furthermore, it is pivotal to further investigate the role of expectancy of rejection in the measurement of rejection sensitivity.

## Figures and Tables

**Table 1:** Sociodemographic characteristics of the sample (*N* = 774).

Age—M (SD)	31.83 (14.45)
Young adults—counts (%)	493 (64.5)
Adults—counts (%)	271 (35.5)
Women—counts (%)	430 (56.3)
Job—counts (%)	
Students	379 (49.6)
Employed	304 (39.8)
Unemployed/retired	70 (9.2)
Marital status—counts (%)	
Not married	485 (63.5)
Married	243 (31.8)
Divorced	23 (3.0)
Widowed	8 (1.0)

**Table 2:** Fit statistics for the concurrent models (estimator: Robust Diagonally Weighted Least Squares).

	Satorra-Bentler Scaled Chi-Square	Root Mean Square Error of Approximation (RMSEA) (90% CI)	Akaike Information Criterion (AIC)	Comparative Fit Index (CFI)	Standardized Root Mean Square Residual (SRMSR)	Degree of freedom
One-factor model [8, 9]	218.72*	0.098 (0.086/0.11)	254.72	0.89	0.060	27
2nd-order factor model	1888.05*	0.14 (0.13/0.14)	1976.05	0.81	0.14	127
Bifactor model	629.45*	0.077 (0.071/0.083)	737.45	0.95	0.067	117

*Significant for *P* < 0.001.

**Table 3:** Standardized factor loadings (measurement errors) for the bifactor model.

Items	Rejection anxiety	Rejection expectancy	General additive factor
*Item 1*—***you ask your parents or another family member for a loan to help you through a difficult financial time ***(*Chiedi ai tuoi genitori (o a un altro familiare) un prestito per superare una fase economica difficile*).			
*(a) How concerned or anxious would you be over whether or not your family would want to help you *(Quanto ti sentiresti in ansia o preoccupato/a per il fatto che tuoi familiari vogliano o non vogliano aiutarti)*? *	0.20	—	0.52
*(b) I would expect that they would agree to help as much as they can *(Mi aspetto che loro vogliano aiutarmi il più possibile).	—	0.50	0.21

*Item 2*—***you approach a close friend to talk after doing or saying something that seriously upset him/her ***(*Ti avvicini per parlare a un tuo amico intimo (o amica intima) dopo che hai fatto o detto qualcosa che l'ha veramente sconvolto/a*).			
*(a) How concerned or anxious would you be over whether or not your friend would want to talk with you *(*Quanto ti sentiresti in ansia o preoccupato/a per il fatto che lui/lei ti voglia o non ti voglia parlare*)*? *	0.44		0.31
*(b) I would expect that he/she would want to talk with me to try to work things out *(*Mi aspetto che lui/lei voglia parlarmi per risolvere le cose*).	—	0.47	0.20

*Item 3*—***you bring up the issue of sexual protection with your significant other and tell him/her how important you think it is ***(*Inizi a dire al tuo partner quanto è importante per te usare misure di protezione durante il rapporto sessuale*).			
*(a) How concerned or anxious would you be over his/her reaction *(*Quanto ti sentiresti in ansia o preoccupato/a per la sua reazione*)*? *	0.12		0.64
*(b) I would expect that he/she would be willing to discuss our possible options without getting defensive *(*Mi aspetto che lui/lei voglia discutere le possibili alternative senza mettersi sulla difensiva*).	—	0.49	0.27

*Item 4*—***you ask your supervisor for help with a problem you have been having at work ***(*Chiedi al tuo supervisore un aiuto per risolvere un problema che stai avendo nel tuo lavoro*).			
*(a) How concerned or anxious would you be over whether or not the person would want to help you *(*Quanto ti sentiresti in ansia o preoccupato/a per il fatto che lui/lei voglia o non voglia aiutarti*)*? *	0.40		0.51
*(b) I would expect that he/she would want to try to help me out *(*Mi aspetto che lui/lei voglia provare ad aiutarmi*).	—	0.41	0.31

*Item 5*—***after a bitter argument, you call or approach your significant other because you want to make up ***(*Dopo una aspra discussione, ti avvicini per parlare (o telefoni) al tuo partner perchè vuoi risolvere il problema che si è creato tra di voi*).			
*(a) How concerned or anxious would you be over whether or not your significant other would want to make up with you *(*Quanto ti sentiresti in ansia o preoccupato/a per il fatto che lui/lei voglia o non voglia risolvere il problema che si è creato*)*? *	0.62		0.49
*(b) I would expect that he/she would be at least as eager to make up as I would be *(*Mi aspetto che lui/lei sia desideroso/a quanto me di risolvere il problema che si è creato tra di noi*).	—	0.62	0.30

*Item 6*—***you ask your parents or other family members to come to an occasion important to you ***(*Chiedi ai tuoi genitori (o a un altro familiare) di essere presenti per una occasione importante per te*).			
*(a) How concerned or anxious would you be over whether or not they would want to come *(*Quanto ti sentiresti in ansia o preoccupato per il fatto che loro vogliano o non vogliano venire*)*? *	0.09		0.77
*(b) I would expect that they would want to come *(*Mi aspetto che loro vogliano essere presenti*).	—	0.60	0.37

*Item 7*—***at a party, you notice someone on the other side of the room that you'd like to get to know, and you approach him or her to try to start a conversation ***(*A una festa noti una persona dall'altra parte della stanza che vorresti conoscere e l'avvicini per provare ad iniziare una conversazione*).			
*(a) How concerned or anxious would you be over whether or not the person would want to talk with you *(*Quanto ti sentiresti in ansia o preoccupato/a per il fatto che questa persona voglia o non voglia parlare con te*)*? *	0.48		0.42
*(b) I would expect that he/she would want to talk with me *(*Mi aspetto che lui/lei voglia parlare con me*).	—	0.33	0.39

*Item 8*—***lately you've been noticing some distance between yourself and your significant other, and you ask him/her if there is something wrong ***(*Ultimamente hai notato che si è creata una certa distanza tra te e il tuo partner e gli/le chiedi se c'è qualcosa che non va*).			
*(a) How concerned or anxious would you be over whether or not he/she still loves you and wants to be with you *(*Quanto ti sentiresti in ansia o preoccupato/a per il fatto che lui/lei ti ami ancora o no e voglia o non voglia stare con te?)? *	0.68		0.38
*(b) I would expect that he/she will show sincere love and commitment to our relationship no matter what else may be going on *(M*i aspetto che lui/lei mi mostri un amore sincero e desideri continuare la nostra relazione nonostante i problemi che possano esserci*).	—	0.56	0.25

*Item 9*—***you call a friend when there is something on your mind that you feel you really need to talk about ***(*Telefoni a un tuo amico/a perchè hai qualcosa di cui hai veramente bisogno di parlare*).			
*(a) How concerned or anxious would you be over whether or not your friend would want to listen *(*Quanto ti sentiresti in ansia o preoccupato/a per il fatto che lui/lei voglia o non voglia starti ad ascoltare*)*? *	0.09		0.75
*(b) I would expect that he/she would listen and support me *(*Mi aspetto che lui/lei voglia starmi a sentire e darmi supporto*).	—	0.56	0.39

**Table 4:** Descriptive statistics (*N* = 774).

	M (SD)	Cronbach alpha	Interitem mean correlation	McDonald's omega H	McDonald's omega S
ARSQ general factor	2.69 (0.74)	0.82	0.20	0.28	—
Rejection anxiety	3.03 (1.02)	0.82	0.34	0.05	0.18
Rejection expectancy	2.36 (0.82)	0.78	0.28	0.10	0.34
BDI-II	9.30 (8.20)	0.89	0.29	—	—
BAI	34.38 (10.89)	0.92	0.35	—	—
BHS	4.89 (4.08)	0.85	0.22	—	—

ARSQ: Adult Rejection Sensitivity Questionnaire; BDI-II: Beck Depression Inventory-II; BAI: Beck Anxiety Inventory; BHS: Beck Hopelessness Scale.

**Table 5:** Correlations between measures (*N* = 772).

		1	2	3	4	5
1	ARSQ general factor					
2	Rejection anxiety	0.84∗∗				
3	Rejection expectancy	0.75∗∗	0.27∗∗			
4	BDI-II	0.41∗∗	0.40∗∗	0.23∗∗		
5	BAI	0.31∗∗	0.31∗∗	0.16∗∗	0.60∗∗	
6	BHS	0.38∗∗	0.32∗∗	0.28∗∗	0.50∗∗	0.29∗∗

**Correlation is significant at the 0.01 level (2-tailed).

ARSQ: Adult Rejection Sensitivity Questionnaire; BDI-II: Beck Depression Inventory-II; BAI: Beck Anxiety Inventory; BHS: Beck Hopelessness Scale.
